# 2, 3-Dihydro-3β-methoxy Withaferin-A Lacks Anti-Metastasis Potency: Bioinformatics and Experimental Evidences

**DOI:** 10.1038/s41598-019-53568-6

**Published:** 2019-11-22

**Authors:** Anupama Chaudhary, Rajkumar S. Kalra, Vidhi Malik, Shashank P. Katiyar, Durai Sundar, Sunil C. Kaul, Renu Wadhwa

**Affiliations:** 1 DAILAB, DBT-AIST International Center for Translational & Environmental Research (DAICENTER), National Institute of Advanced Industrial Science & Technology (AIST), Tsukuba, 305 8565 Japan; 2 Department of Biochemical Engineering & Biotechnology, DAILAB, Indian Institute of Technology (IIT)-Delhi, Hauz Khas, New Delhi, 110 016 India

**Keywords:** Cell invasion, Drug development

## Abstract

Withaferin-A is a withanolide, predominantly present in Ashwagandha (*Withania somnifera*). It has been shown to possess anticancer activity in a variety of human cancer cells *in vitro* and *in vivo*. Molecular mechanism of such cytotoxicity has not yet been completely understood. Withaferin-A and Withanone were earlier shown to activate p53 tumor suppressor and oxidative stress pathways in cancer cells. 2,3-dihydro-3β-methoxy analogue of Withaferin-A (3βmWi-A) was shown to lack cytotoxicity and well tolerated at higher concentrations. It, on the other hand, protected normal cells against oxidative, chemical and UV stresses through induction of anti-stress and pro-survival signaling. We, in the present study, investigated the effect of Wi-A and 3βmWi-A on cell migration and metastasis signaling. Whereas Wi-A binds to vimentin and heterogeneous nuclear ribonucleoprotein K (hnRNP-K) with high efficacy and downregulates its effector proteins, MMPs and VEGF, involved in cancer cell metastasis, 3βmWi-A was ineffective. Consistently, Wi-A, and not 3βmWi-A, caused reduction in cytoskeleton proteins (Vimentin, N-Cadherin) and active protease (u-PA) that are essential for three key steps of cancer cell metastasis (EMT, increase in cell migration and invasion).

## Introduction

Cancer cell metastasis is a multistep process in which cancer cell acquires higher migration ability, invasive characteristics to permeate the basement membrane/extracellular matrix, access to lymphatic or vascular circulation towards developing secondary tumors at distant sites^1^. Metastatic malignancies have been shown to contribute to about 90% of cancer-related mortalities. Metastatic tumor cells, not only escape conventional radiotherapy, but also show a high level of resistance to a variety of chemotherapeutic combinations^2^. At molecular level, a cancer cell while acquiring metastatic ability, undergoes a drastic remodeling of cytoskeleton and membrane components^3^. Increase in vimentin (Type III intermediate filament protein), N-cadherin (transmembrane glycoprotein) and loss of E-cadherin have been characterized as key molecular alterations during Epithelial-to-Mesenchymal Transition (EMT), a primary step in cancer metastasis. A family of secretory proteases facilitates invasion of tumor cells through extracellular matrix and neighboring tissue compartments^3,4^. Therein, membrane-bound serine protease urokinase-type plasminogen activator (uPA) and matrix metalloproteinase (MMPs) proteases primarily degrade the extracellular matrix and facilitate extravasation of a tumor cells^5^. Process of angiogenesis mediated by secreted mitogens and vascular endothelial growth factor (VEGF), a key angiogenic factor, supports the formation of new blood vessels and promotes endothelial cell proliferation, cell migration, and survival^6^.

Withaferin A (Wi-A), is a potent bioactive withanolide that was first isolated from the roots of Ashwagandha (*Withania somnifera*), an Indian Ayurvedic medicinal herb. In initial investigations, Wi-A was shown to possess anticancer activity through diverse mechanisms including activation of caspase-3 and pERK, inhibition of JNK, Akt and IL6 pro-survival signaling^7,8^, instigation of oxidative stress and DNA damage response (DDR), leading to growth arrest or apoptosis in cancer cells in a dose-dependent manner^9^. Wi-A was shown to induce vimentin aggregation, inhibiting invasion and metastases of the breast cancer cells^10^. Bargagna-Mohan *et al*. showed that Wi-A alters vimentin assembly by covalent modification of its cysteine 328 residue^11^; later it was found to cause vimentin phosphorylation^12^. More recently, some studies showed that the treatment with Wi-A containing root extract inhibited metastases and EMT process^13,14^. We had earlier demonstrated that Wi-A and Withanone inhibit cancer cell migration and invasion by downregulating heterogeneous nuclear ribonucleoprotein-K (hnRNP-K), VEGF and MMPs proteins^15^.

2,3-dihydro-3β-methoxy withaferin-A (3βmWi-A) is a natural and structurally close Wi-A analogue, having substitution of β-methoxy group at position 3 of Wi-A ergostane ring^16^. Structural analogue of natural compounds, having chemical modification i.e. methylation, acetylation or hydroxylation at key moieties has been shown to affect their bioactivity, underlining structure-function association^17^. More precisely, alkali analogues of organic steroid compounds have been shown to attain improved potency^18,19^. Markedly, 10-methoxy derivative of Camptothecin, was shown to induce anti-angiogenic response^20^. Moreover, dimethoxy substitution to Brartemicin, a trehalose-based metastasis inhibitor, was shown to improve potency in murine colon carcinoma model^21^. Similarly, cis-resveratrol methylated analogs were shown to augment anti-tumor potency in metastatic mouse melanoma cells^22^. 2-methoxyestradiol, a natural estrogen analogue was characterized to be a potent anti-angiogenic compound^23^.

In light of these findings, we had earlier characterized the activities of Wi-A and its methoxy analogue 3βmWi-A. Unlike Wi-A, 3βmWi-A was found to be safe and well-tolerated at even higher concentration *in vitro*^16^. On contrary to Wi-A, 3βmWi-A was found to be inert and lacked cytotoxicity, and shown to promote survival of normal human cells exposed to various stress conditions^24^. Consistently and the fact that some natural compounds at nontoxic doses have been shown to possess anti-metastasis activity^25,26^, we investigated the effect of 3βmWi-A on cancer cell migration using bioinformatics and molecular assays in cultured cells. We report that whereas Wi-A binds to vimentin and hnRNP-K and modulate VEGF and MMPs signaling, 3βmWi-A lacked these activities.

## Results

### Wi-A, but not 3βmWi-A, inhibited cancer cell migration and invasion

**Figure 1:**
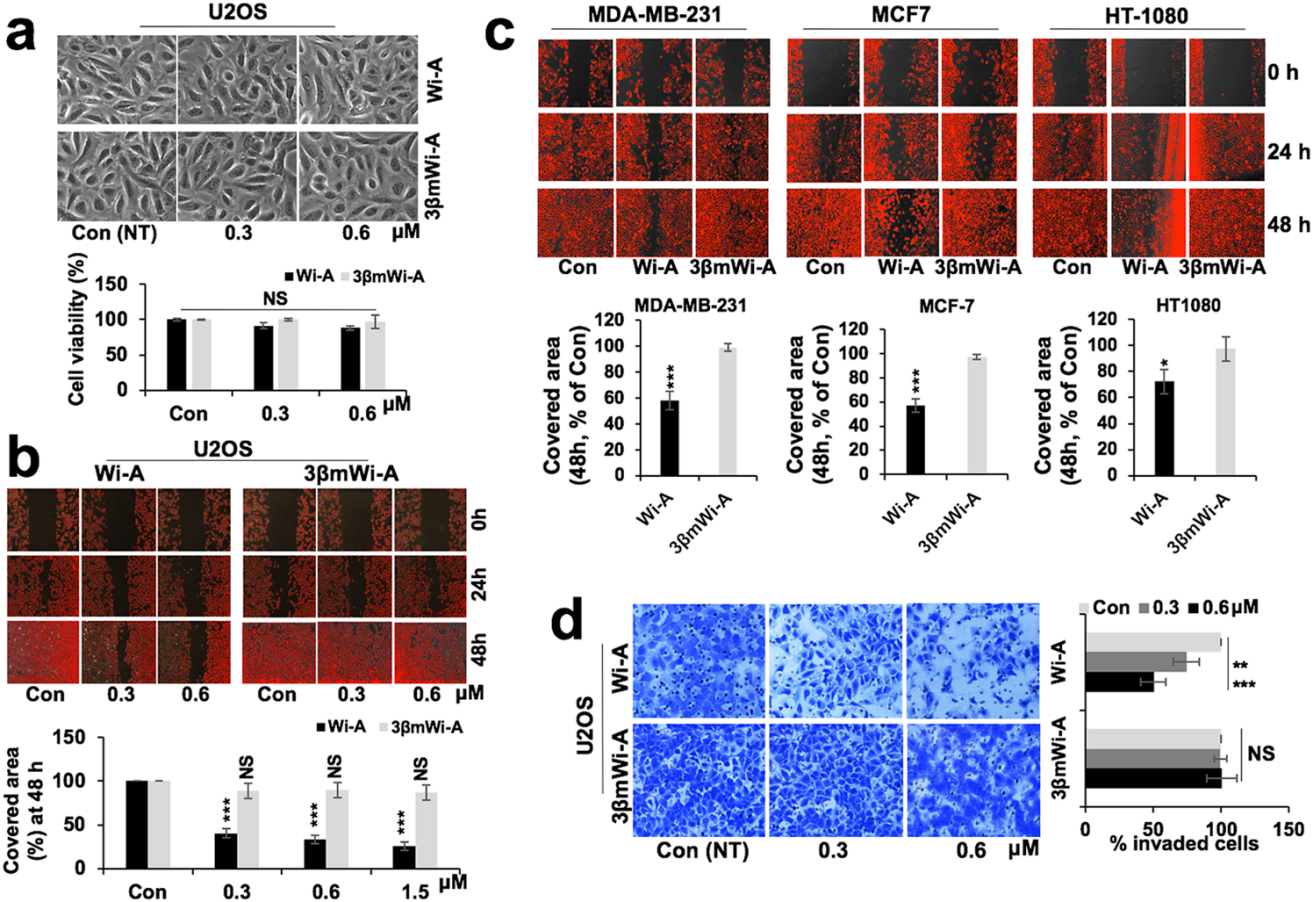
Wi-A, and not 3βmWi-A, inhibited cancer cell migration and invasion. (**a**) Representative phase contrast images showing cell morphology of control and Wi-A and 3βmWi-A (sub-toxic concentrations; 0.3 and 0.6 μM) treated cells. Quantitation of cell viability assay in U2OS control and treated cells at 48 h is shown. (**b**) Representative images from wound healing assay showing scratched area in control and Wi-A and 3βmWi-A treated U2OS cells at 0 h, 24 h and 48 h time points; while its quantitation showing percentage of gap filled at 48 h time point is shown in the lower panel. (**c**) Representative images from wound healing assay performed in metastatic MDA-MB-231, MCF7 and HT-1080 cell lines showing scratched area in control and 0.6 μM Wi-A and 3βmWi-A treated sets at 0 h, 24 h and 48 h time points; quantitation (lower panel) showing percentage covered area in Wi-A and 3βmWi-A treated cells (taking control as 100%) at 48 h. (**d**) Representative images from Matrigel invasion assay showing invaded crystal violet stained cells in control, Wi-A and 3βmWi-A treated cells. Quantitation on the right, showing percentage of invaded cells in control, Wi-A and 3βmWi-A treated cells.

In order to investigate the effect of Wi-A and 3βmWi-A on cancer cell migration, we used their sub-toxic doses (0.3 and 0.6 μM)^16,27^. As shown in Fig. 1a, analyses of cell phenotype and viability showed no significant effect on U2OS (osteosarcoma) cells. In wound-healing assay, we found that whereas Wi-A inhibited U2OS cell migration at even the low dose (0.3 μM), 3βmWi-A did not cause any difference with respect to the control at both doses used (Fig. 1b). Quantitation of these data (Fig. 1b) showed dose-dependent and significant delay in migration of cells when treated with Wi-A, but not 3βmWi-A. To confirm that the anti-migration effect of Wi-A was not due to its cytotoxicity, time-lapse observations in control, Wi-A and 3βmWi-A (0.3 μM) treated GFP-labeled U2OS cells revealed that Wi-A, but not 3βmWi-A, delayed U2OS cell migration (data not shown); while no cell death was observed. We also confirmed the effect on three other metastatic cancer cell lines (MDA-MB-231, MCF-7 and HT-1080). As shown in Fig. 1c (photomicrographs), whereas sub-toxic doses of Wi-A caused significant delay in migration of all three cell types, 3βmWi-A remained inert. Quantitation of the data (Fig. 1c) showed Wi-A, not 3βmWi-A possesses significant anti-migratory activity. We also performed quantitative Matrigel invasion analyses that revealed a potent anti-invasion effect of Wi-A, but not of 3βmWi-A, both at 0.3 and 0.6 μM concentrations (Fig. 1d). Furthermore, a dose dependent effect (30 to 50% decrease) on invasiveness (both 0.3 and 0.6 μM) was observed in Wi-A treated U2OS cells. Conversely, 3βmWi-A did not exhibit anti-invasion activity. Therefore, these results suggested that Wi-A, and not 3βmWi-A, inhibited cancer cell migration and invasion (Fig. 1).

### Molecular docking of Wi-A and 3βmWi-A with Vimentin

In light of the information that Wi-A caused vimentin aggregation resulting in abrogation of invasion and metastases of the breast cancer cells^10–12^, we examined the interactions of these two molecules with vimentin by bioinformatics and molecular docking (MD) tools. The computationally modelled structure of vimentin dimer and tetramer was retrieved and vimentin monomer coordinates were extracted from dimer structure. MD simulation of vimentin modelled structure from residue 79–406 (monomer, dimer and tetramer) was carried out for 50 ns. The average representative structure of Vimentin was selected from stable simulation trajectories for each case. The RMSD of each structure was also analyzed to treat it as a control (Fig. S1a). The RMSD comparison of Vimentin monomer with its dimeric and tetrameric forms showed that Vimentin monomer is highly unstable whereas dimer and tetrameric forms have comparable and stable RMSD fluctuations. The interaction of Wi-A and 3βmWi-A was examined near Cys328 residue of Vimentin using molecular docking and simulation tools. Cys328 residue plays important role in reorganization of Vimentin network in response to oxidants as well as Vimentin polymerization by interacting with Zinc^28^. Interaction of any compounds at this critical residue may affect polymerization and assembly of Vimentin. Binding affinity and interactions formed by Wi-A and 3βmWi-A at the binding site of Vimentin monomer, dimer and tetramer are listed in Table 1. MM-GBSA was also used to find ligand binding affinity in docked complexes before and after MD simulations (Table 1).

**Table 1:** Details of interactions formed by withanolides at protein binding site along with calculated binding energy of protein-ligand complexes and their docking scores.

Protein	Withanolide	Docking Score (Kcal/mol)	MM-GBSA Binding energy (Kcal/mol)		Interactions at the binding site	
			Before MD Simulation	After MD Simulation	Hydrogen Bonds	Hydrophobic Interactions
Vimentin monomer	Wi-A	−1.77	−98.25	−49.61	Ser325	Gln324, Leu326, Leu333, Thr336, Leu340 and Asn337
	3βmWi-A	−2.76	−29.51	−35.44	—	Gln324, Leu326, Val330, Leu333, Lys334, Leu340, Glu341 and Asn337
Vimentin dimer	Wi-A	−2.462	−32.74	−37.59	Chain A: Arg320	Chain A: Tyr319, Val323, Gln324 and Thr327Chain B: Tyr 319 and Gln322
	3βmWi-A	−1.710	−39.43	−61.00	Chain B: Thr327	Chain A: Gln322, Ser325, Leu326 and Leu333 Chain B: Val323, Thr327, Val330 and Asn337
Vimentin tetramer	Wi-A	−2.264	−25.17	−93.51	—	Chain A: Glu315, Tyr319, Gln322, Ser325, Leu326 and Glu329Chain B: Val323, Thr327 and Val330
	3βmWi-A	−2.885	−29.85	−78.48	Chain A: Gln322	Chain A: Glu315 and Ser325Chain B: Ser316, Tyr319, Arg320, Val323 and Thr327

**Figure 2:**
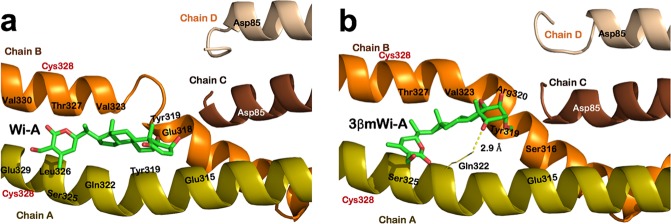
Molecular docking of Wi-A and 3βmWi-A with Vimentin. Molecular interactions of vimentin tetramer at the binding site around Cys328 with Wi-A (**a**) and 3βmWi-A (**b**).

Binding affinity of ligands reported by MM-GBSA were significant, hence, we proceeded with MD simulation of these complexes to investigate stability of interactions predicted at the binding site. Both molecules were found to be stable at the binding site of all forms of Vimentin, interacting with almost same residues but with slightly different orientations (Fig. 2a,b and Fig. S2a–d). Wi-A and 3βmWi-A did not show any interaction with Cys328, however, both interacted with its neighboring residues and could interfere with interaction of other entities with Cys328, thereby affecting polymerization and reorganization of Vimentin filaments. Next, we examined the effect of Wi-A and 3βmWi-A on stability of protein structure. RMSD computation of Vimentin monomer (Control) and in complex with Wi-A and 3βmWi-A showed that ligand binding around Cys328 increased the stability of Vimentin monomer structure, which was comparable to its dimeric and tetrameric forms (Fig. S1b). Of note, we found that Wi-A added more stability to Vimentin monomer (RMSD fluctuations in range of 20–30 Å) as compared to 3βmWi-A (that is 30–40Å). In line with this, the MM-GBSA calculated binding affinity of Wi-A (−49.61 Kcal/mol), was stronger than 3βmWi-A (−35.44 Kcal/mol). We also found that Wi-A formed both hydrophobic and hydrogen bond interactions, while 3βmWi-A interacted solely by hydrophobic interactions and did not form any hydrogen bonds (Fig. S2a,b). For Vimentin dimer and tetramer, RMSD fluctuations of Vimentin (control), Vimentin-Wi-A complex and Vimentin-3βmWi-A complex were comparable (Fig. S1c). These data suggested that Wi-A and 3βmWi-A may not affect stability of Vimentin dimer and tetramer. In case of Vimentin dimer, 3βmWi-A showed stronger binding efficiency by laterally interacting with both chains of dimer (binding energy of −61 Kcal/mol). On the other hand, Wi-A showed longitudinal interactions with chain A only (binding energy of −37.59 Kcal/mol) (Fig. S2c,d). In addition, both Wi-A and 3βmWi-A formed hydrophobic interaction with Gln322, a conserved residue found in the highly conserved domain protein family of intermediate filament (Fig. S3)^29^. Similarly, in case of Vimentin tetramer both Wi-A and 3βmWi-A showed binding at the same site forming interactions with almost same residues with slightly different binding affinity of −93.51 and −78.48 Kcal/mol, respectively (Fig. 2a,b). Both Wi-A and 3βmWi-A interacted with conserved residue Gln322. However, we found that Wi-A caused local disruption of helical structure at the binding site while no such effect was observed in case of 3βmWi-A. Such configurations are predicted to affect interaction of Vimentin with metal ions (Zn) for formation of Vimentin octamer^28^. These data suggested that both Wi-A and 3βmWi-A can interact with Vimentin monomeric, dimeric and tetrameric forms. Wi-A, however, showed high binding affinity for and also caused disruption of secondary structure at the binding site of vimentin tetramer. Hence, it was predicted that Wi-A, but not 3βmWi-A, targets Vimentin causing anti-migration and anti-metastatic effects.

**Figure 3:**
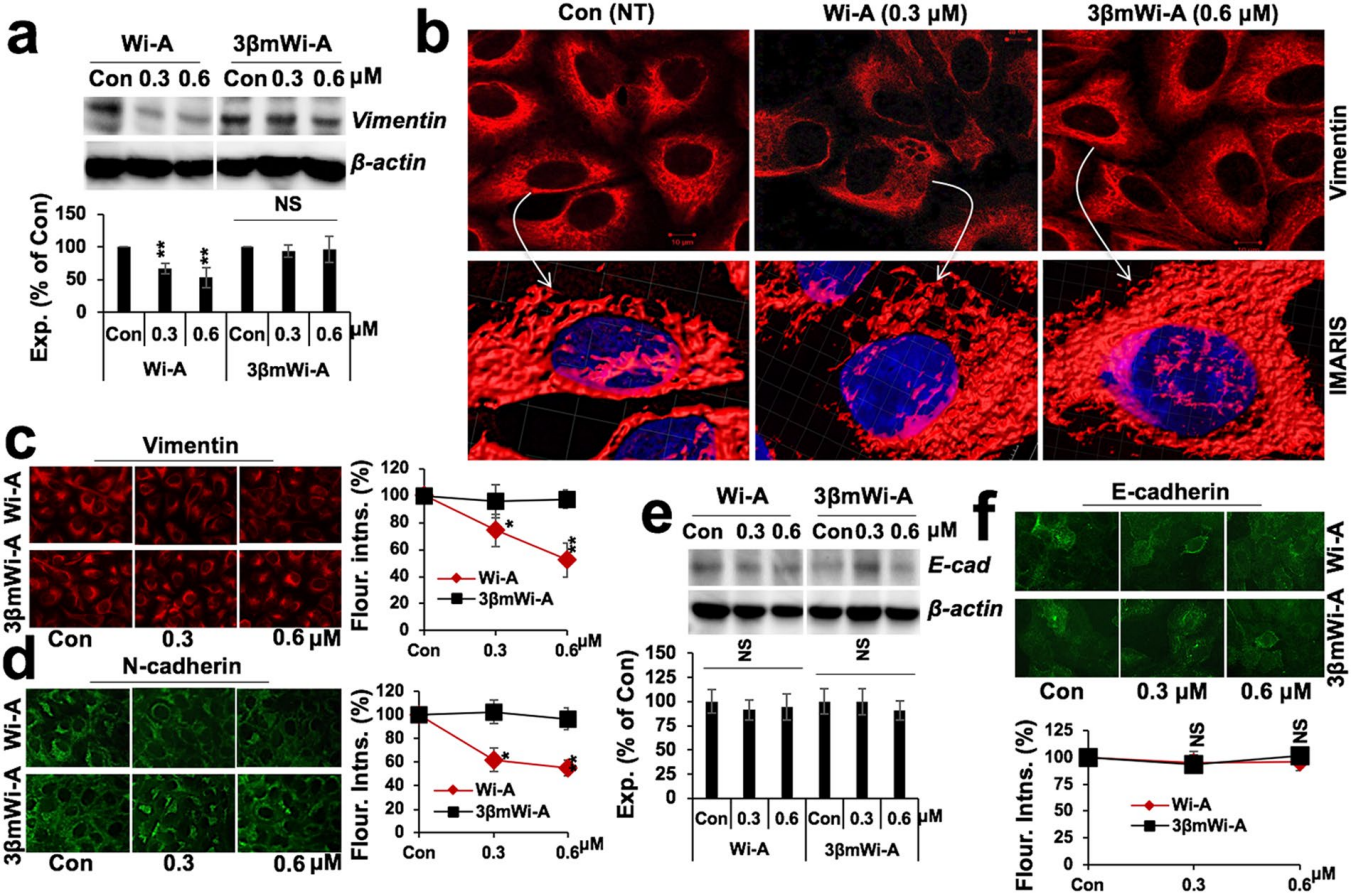
Wi-A, and not 3βmWi-A, affected Vimentin and N-Cadherin. (**a**) Immunoblotting (upper panel) and its quantitation (lower panel) showing Vimentin expression levels in control, 0.3 μM, and 0.6 μM Wi-A and 3βmWi-A (cropped blot omitting their 1.5, and 3 μM toxic doses) treated U2OA cancer cells. (**b**) Immunostaining showing vimentin expression and distribution in treated cells. Inset image (lower panel) (marked by white arrow), showing vimentin aggregates in 0.6 μM Wi-A cells, though no similar aggregates were observed in control and 3βmWi-A treated cells. (**c,d**) Vimentin and N-cadherin immunostaining showing their expression levels in control and 0.3, 0.6 μM Wi-A and 3βmWi-A treated cells; their quantitation is shown on the right. (**e**) Immunoblot showing unaltered E-cadherin expression in Wi-A and 3βmWi-A treated cells (cropped blot omitting their 1.5, and 3 μM toxic doses); quantitation of its expression is shown in the lower panel. (**f**) Immunostaining showing unaltered E-cadherin expression in treated cells; quantitation shown at the bottom.

We next performed molecular analyses to confirm the above results. As shown in Fig. 3a, we found significant decrease in Vimentin, a key intermediate filament protein involved in cell migration, in Wi-A, but not 3βmWi-A, treated cells. We, next, analyzed the localization and assembly of Vimentin in control and treated cells. Whereas Wi-A (0.6 μM) treated cells showed distinct Vimentin aggregation (Fig. 3b), there was no difference in response to 3βmWi-A treatment. The results were confirmed with high resolution confocal microscopy (Fig. 3b). Dose dependent effect of Wi-A and 3βmWi-A, and analysis of Vimentin expression by immunostaining further revealed its significant reduction in Wi-A treated cells, while its levels remain unchanged in response to 3βmWi-A treatment (Fig. 3b,c). Based on these data and Figs. 1 and 2, it was confirmed that whereas Wi-A targeted Vimentin protein, 3βmWi-A lacks such activity. The data was consistent to an earlier report that showed Vimentin as a target of Wi-A^10^. In order to get further insights to the differential bio-activities of Wi-A and 3βmWi-A, we examined their effect on the expression of N-cadherin and E-cadherin, two key mesenchymal and epithelial cell markers. As shown in Fig. 3d, Wi-A, but not 3βmWi-A, caused decrease in N-cadherin in dose-dependent manner (Fig. 3d). In contrast, there was no change in the level of E-cadherin as validated by immunoblotting (Fig. 3e) and immunostaining (Fig. 3f), respectively. These data suggested that Wi-A has potent anti-migratory activities, while 3βmWi-A lacks such effect.

### Interactions of Wi-A and 3βmWi-A with the KH3 domain of hnRNP-K and functional consequences

**Figure 4:**
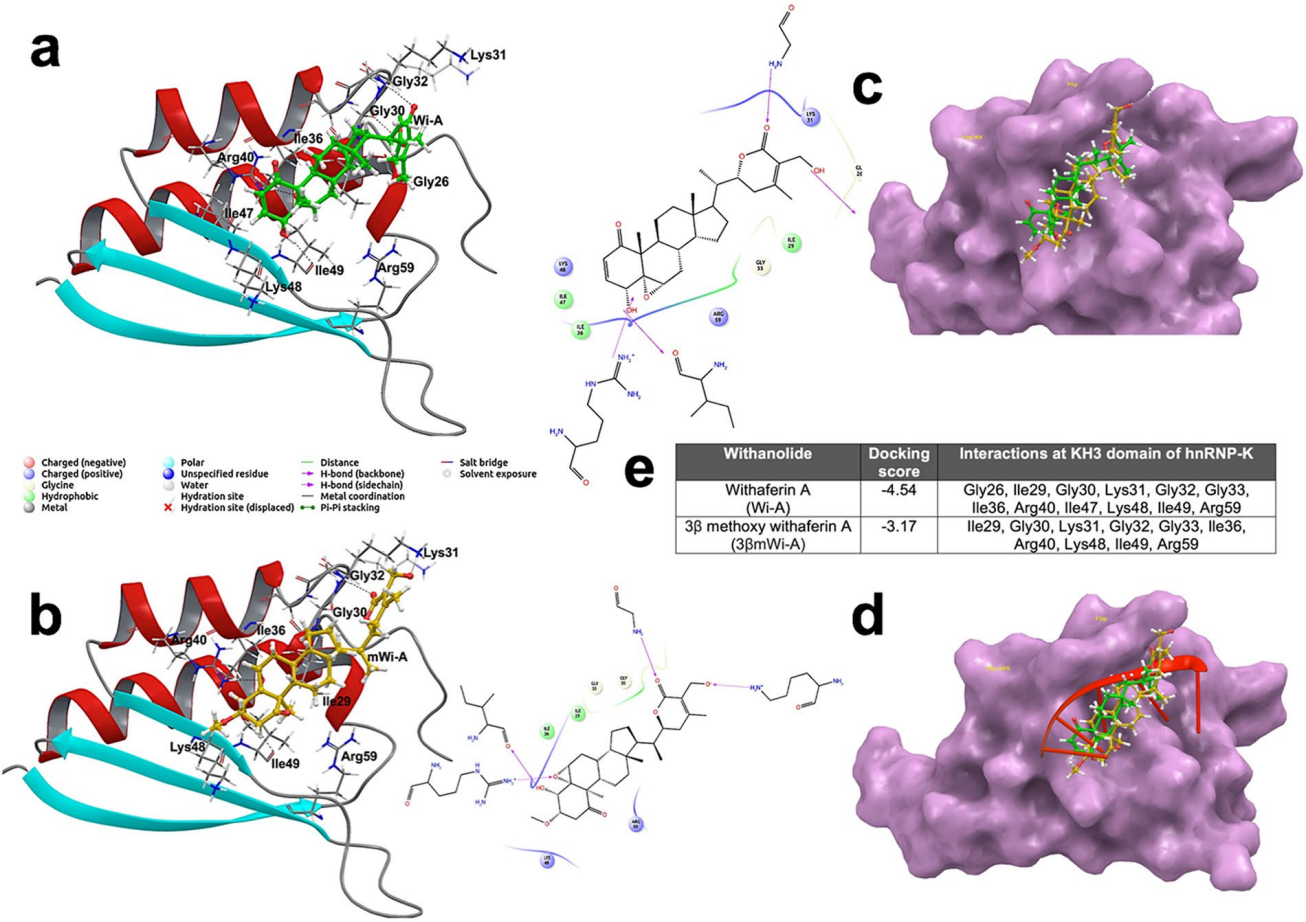
Interactions of Wi-A and 3βmWi-A with the KH3 domain of hnRNP-K. (**a**) Molecular modeling showing binding of Wi-A (green) at the DNA binding interface of hnRNP-K. (**b**) Molecular binding of 3βmWi-A (yellow) at the DNA binding interface of hnRNP-K. (**c**) Table showing details of docking score and predicted molecular interactions of hnRNP-K with Wi-A & 3βmWi-A. (**d**) Superimposed docking conformations of Wi-A (green) and 3βmWi-A (yellow), bound at KH3 domain of hnRNP-K. (**e**) Superimposed Wi-A (green), 3βmWi-A (yellow) and ssDNA (red) at the DNA binding KH3 domain of hnRNP-K.

**Figure 5:**
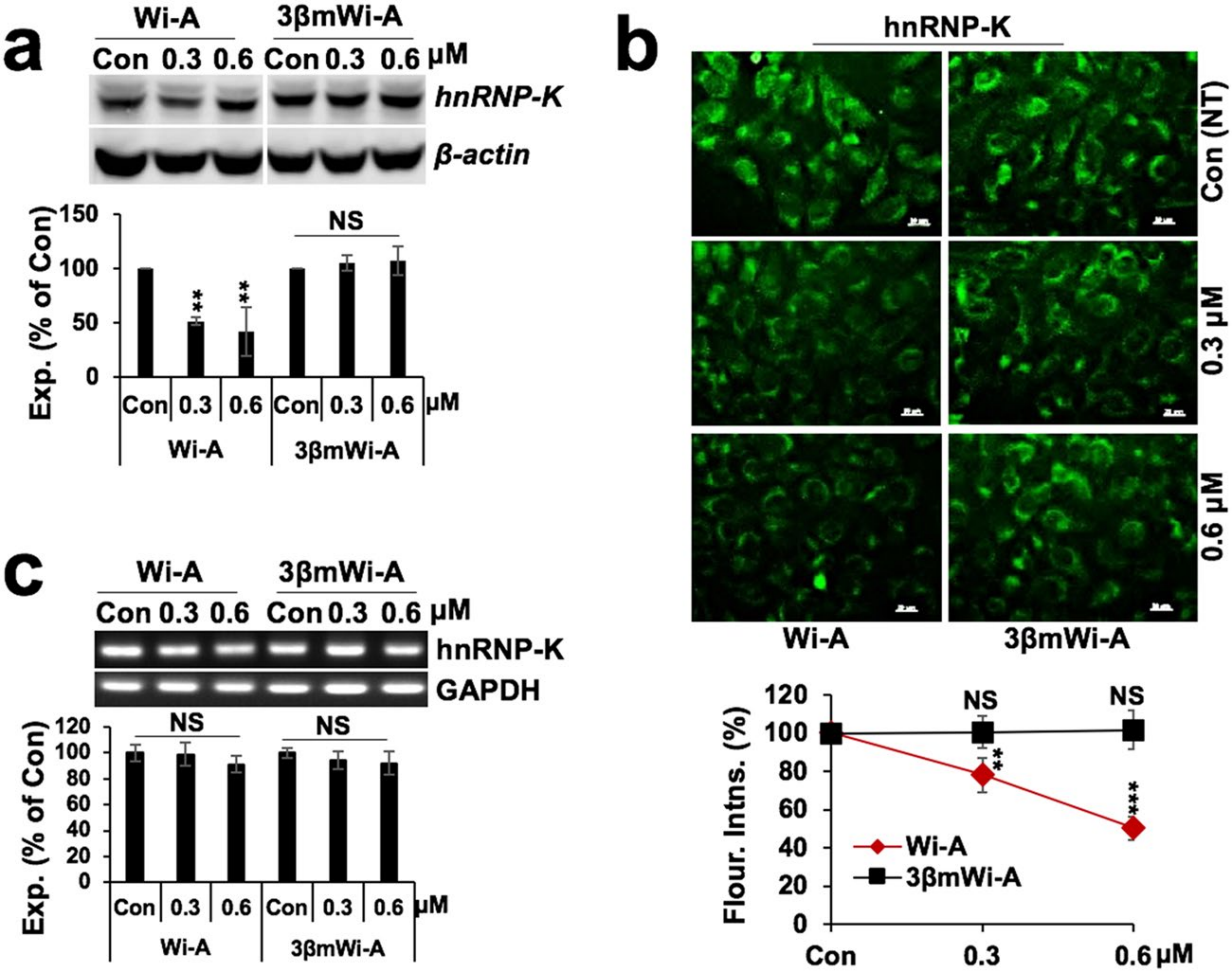
Wi-A, and not 3βmWi-A, inhibited hnRNP-K at protein levels. (**a**) Immunoblot showing hnRNP-K expression in control, 0.3 μM, and 0.6 μM Wi-A and 3βmWi-A (cropped blot omitting their 1.5, and 3 μM toxic doses) treated U2OS cancer cells; quantitation of its expression is shown in the lower panel. (**b**) Immunostaining showing hnRNP-K expression in the treated cells; quantitation of its expression is shown at the bottom. (**c**) RT-PCR showing hnRNP-K transcript in the treated cells; quantitation of its expression is shown in the lower panel.

In light of the above findings and our earlier report that Wi-A targets hnRNP-K protein^15^, we next examined the effect of 3βmWi-A on hnRNP-K activity. Molecular docking and simulation analyses using KH3 domain of hnRNP-K that has been shown to bind to ssDNA revealed strong binding strength of Wi-A (docking score Wi-A −4.54 Kcal/mol) as compared to 3βmWi-A (docking score 3βmWi-A −3.17 kcal/mol) (Fig. 4a,b,e). Although the two withanolides shared similar configuration and surrounding residues, analysis of their superimposed interactions with hnRNP-K 3D-structure demonstrated that β-methoxy group of 3βmWi-A at C3 position caused steric hindrance and hence decreased its binding strength as compared to Wi-A (Fig. 4c,d). As shown in Fig. 4a,b,e, the superimposed images on the binding of Wi-A and 3βmWi-A with the ssDNA-binding interface revealed weaker docking capability of 3βmWi-A as compared to Wi-A. Following these results, we next examined the expression of hnRNP-K in control and treated cells. As shown in Fig. 5a, immunoblotting revealed dose-dependent decrease in its protein levels in Wi-A, but not 3βmWi-A, treated cells. Furthermore, immunostaining analysis also revealed a decrease in hnRNP-K expression levels in Wi-A treated cells (Fig. 5b). Of note, no change in hnRNP-K transcript levels were observed in Wi-A or 3βmWi-A treated cells (Fig. 5c, below).

### Wi-A, but not 3βmWi-A, inhibits hnRNP-K-VEGF signaling

**Figure 6:**
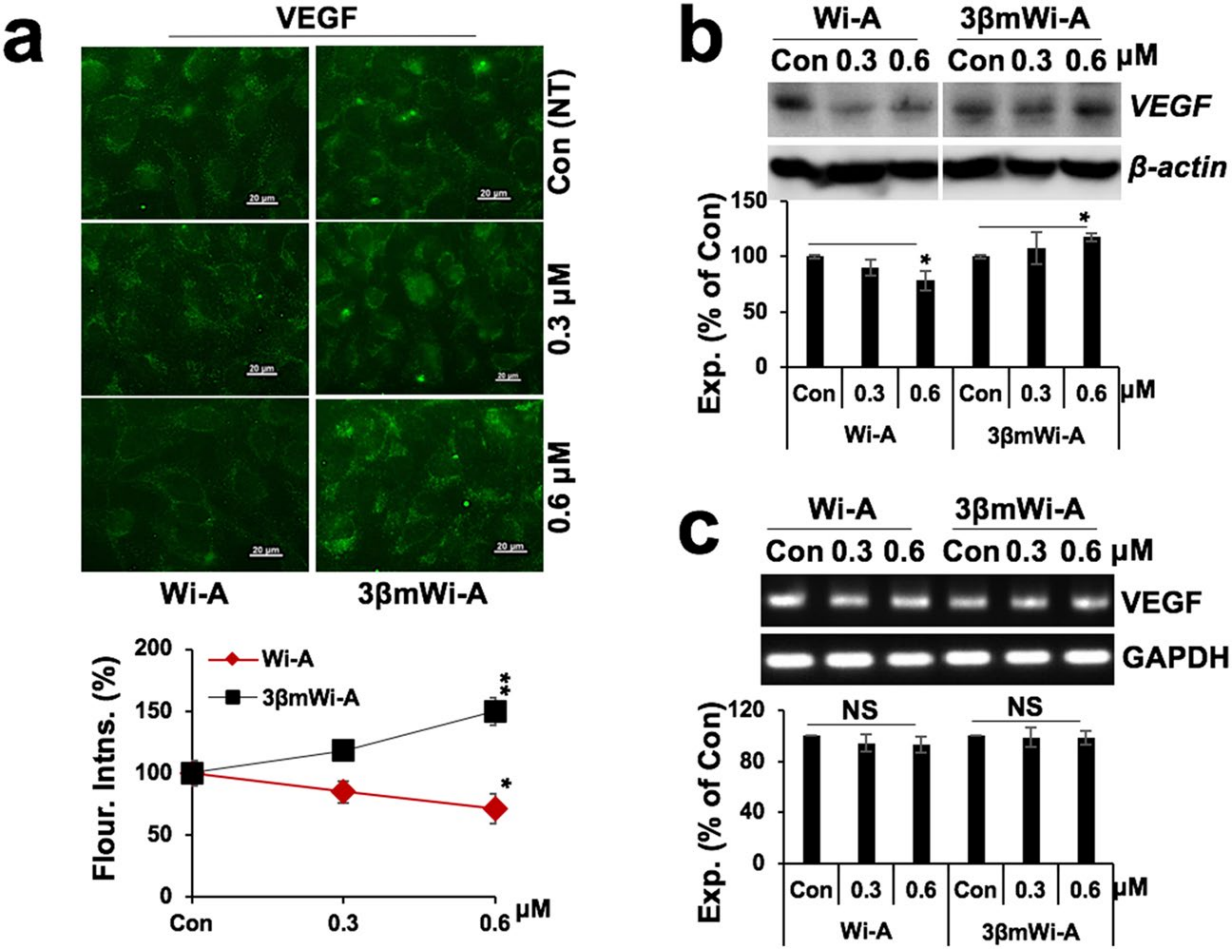
Wi-A, and not 3βmWi-A, reduced the level of VEGF expression. (**a**) Immunostaining showing VEGF expression in control and 0.3, 0.6 μM Wi-A and 3βmWi-A treated U2OS cancer cells; quantitation of its expression is shown in the lower panel. (**b**) Immunoblot showing VEGF expression in Wi-A and 3βmWi-A treated cells (cropped blot omitting their 1.5, and 3 μM toxic doses); quantitation of its expression is shown below. (**c**) RT-PCR showing VEGF transcript in the treated cells; quantitation of its expression is shown in the lower panel.

**Figure 7:**
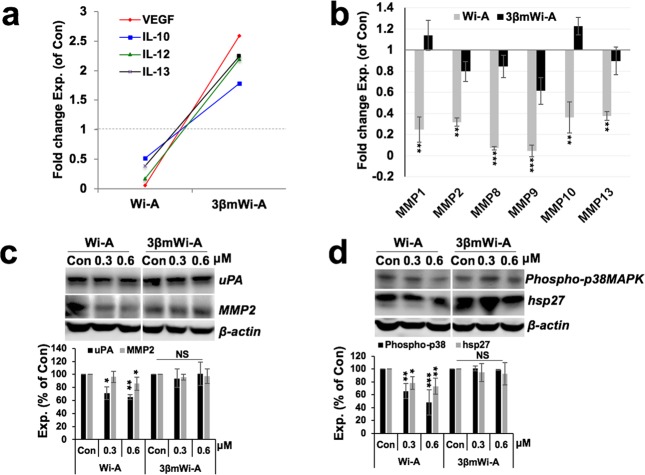
Wi-A, and not 3βmWi-A, inhibits hnRNP-K-VEGF signaling. (**a**) Multiplex immunoarray showing quantitative levels of VEGF and IL-10, 12, 13 pro-metastases secretory proteins in Wi-A and 3βmWi-A treated cells; expression values were normalized with untreated control condition media and represented as fold change. (**b**) MMP Antibody Array showing quantitative expression levels of MMP1, 2, 8, 9, 10 and 13 proteins in the condition media of the Wi-A and 3βmWi-A treated U2OS cells, expression values normalized with untreated control and represented as fold change. (**c**) Immunoblot showing expressions of uPA and MMP2 in Wi-A and 3βmWi-A treated cells (cropped blot omitting their 1.5, and 3 μM toxic doses); quantitation of their expressions is shown in the lower panel. (**d**) Immunoblot showing expressions of phospho-p38MAPK and hsp27 in Wi-A and 3βmWi-A treated cells (cropped blot omitting their 1.5, and 3 μM toxic doses); quantitation of their expressions is shown at the bottom.

In light of the fact that hnRNP-K is a key regulator of VEGF and promotes its mRNA stability and translation^30,31^, we next prompted to examine VEGF level in Wi-A and 3βmWi-A treated cells. As expected, Wi-A, but not 3βmWi-A, treated cells showed decrease in VEGF in immunostaining analyses (Fig. 6a). It was further confirmed by immunoblotting analyses that endorsed decrease in the VEGF protein in Wi-A treated cells only **(**Fig. 6b). Of note, 3βmWi-A treated cells, showed higher level of VEGF protein (Fig. 6a,b). VEGF transcript, on the other hand, showed no difference either in Wi-A or 3βmWi-A treated cells (Fig. 6c) suggesting that Wi-A may essentially affects VEGF at the protein level. Consistent to these results, we next examined the level of secreted VEGF in treated cells by multiplex immunoarray and found that whereas Wi-A caused decrease in secreted VEGF levels, it was increased by more than two-fold in 3βmWi-A treated cells (Fig. 7a). Interestingly, IL-10, IL-12, IL-13, key interleukin factors regulating VEGF, levels also showed decrease in response to Wi-A treatment and increase in 3βmWi-A treatment (Fig. 7a). In order to examine the protein targets of Wi-A relevant to cancer cell invasion and metastases effects, we next performed MMP Antibody array. Untreated U2OS cells were used as negative control. As shown in Fig. 7b, Wi-A treated cells showed remarkable decrease in MMP1, MMP2, MMP8, MMP9, MMP10 and MMP13 expression (Fig. 7b). On the other hand, 3βmWi-A treated cells exhibited a slight and insignificant change in their levels, when normalized with untreated control (Fig. 7b). Expression analyses of VEGF downstream effectors *viz*. u-PA^32^ and MMP2^33^ that play vital role in cancer cell migration, also exhibited their reduced levels in Wi-A, but not 3βmWi-A treated cells (Fig. 7c). The data suggested that Wi-A, consistent to the downregulation of hnRNPK-VEGF signaling, suppressed expression of several key MMPs/proteases involved in cell invasion and metastases, wherein 3βmWi-A remained largely ineffective. Also, we examined VEGF downstream targets phospho-p38MAPK and hsp27, two key VEGF markers promoting cancer cell survival during metastases/angiogenesis. As shown in Fig. 7d, a dose-dependent decrease in phospho-p38MAPK and hsp27 proteins was observed in Wi-A, but not in 3βmWi-A treated cells.

## Discussion

Wi-A and 3βmWi-A are natural co-occurring withanolides, isolated from Ashwagandha. 3βmWi-A, with the substitution of a β-methoxy group at Wi-A parental ring is suggested to be a natural Wi-A analogue. Wi-A is potent bioactive compound, and has been shown to inhibit metastases and EMT process *in vitro* and *in vivo* models essentially by promoting Vimentin aggregation^12–16^ and regulating hnRNP-K function^17^. With evident anti-metastatic potency of Wi-A, and the concept that the alkali analogues retain augmented bioactivities^18,19^; we investigated the activities of 3βmWi-A on cancer cell migration at cellular and molecular levels. Taking the sub-toxic concentrations that showed no effect on cell viability, we found Wi-A, but not 3βmWi-A, inhibited cancer cell migration and invasion. By molecular docking and dynamic simulation studies, Wi-A was predicted to possess greater binding affinity to Vimentin and tetramer than 3βmWi-A. It also caused local disruption of helical structure at Vimentin tetramer binding site. Consistently, Wi-A, but not 3βmWi-A, led to Vimentin aggregation. Wi-A treated cells showed decrease in Vimentin, N-cadherin and E-cadherin proteins, signifying compromised cell migration and invasion^34^. Wi-A was earlier shown to target hnRNP-K, a key regulator of cell migration and angiogenesis^15,30^. Inputs from *in silico* analysis confirmed a stronger binding of Wi-A, over 3βmWi-A, at the hnRNPK’s KH3 domain i.e. ssDNA binding domain. It could lead to instability and decrease in hnRNP-K and its downstream effectors including VEGF and MMPs leading to compromised cell migration^15,32,35,36^. Reduced levels of MMPs, uPA proteases and VEGF targets *viz*. phospho-p38MAPK^37^, heat shock protein 27 (HSP27) that promote cancer cell migration^37–39^, were indeed found in Wi-A, but not 3βmWi-A, treated cells. Taken together, we demonstrate that 3βmWi-A lacked cytotoxicity to cancer cells and showed no effect on cell migration, invasion and angiogenic characteristics. Wi-A was found to affect the Vimentin assembly, yielding its decrease in treated cells; 3βmWi-A was ineffective. Wi-A, as compared to 3βmWi-A, also showed stronger binding to hnRNP-K. Additionally, it caused decrease in other key regulators of cell migration, invasion and metastasis, *viz*, MMPs and VEGF, u-PA, Hsp27 and p38MAPK. Taken together, we found that in contrast to Wi-A and the anticipation that alkali analogues may exhibit stronger potency, 3βmWi-A lacked anti-metastatic activity. Hence, structural-function predictions of natural compounds warrant careful experimental validation before their recruitment in disease preventive or therapeutic avenues.

## Methods

### Cell culture

Osteosarcoma (U2OS; U2OS-GFP), fibrosarcoma (HT-1080) and breast adenocarcinoma (MBA-MB-231, MCF-7) cell lines were procured from Japanese Collection of Research Bioresources Cell Bank (JCRB), Tokyo, and cultured in Dulbecco’s Modified Eagle’s Medium (DMEM; Wako, Japan) supplemented with 10% Fetal Calf Serum (FCS) and 1% antibiotics in a humidified incubator containing 5% CO_2_ at 37 °C as described earlier^40^.

### Cell viability assay

5 × 10^3^ cells were seeded in a 96-well plate and allowed to adhere in next 24 h. On the following day, cells were treated with 0.3, 0.6 μM of Wi-A and 3βmWi-A for next 48 h. Tetrazolium dye 3-(4,5-dimethylthiazol-2-yl)−2,5-diphenyltetrazolium bromide (MTT reagent, Invitrogen, Life technologies), was used to determined viability of both control and Wi-A, 3βmWi-A treated cells, as described earlier in details^16^.

### Immunoblotting

Cells pellets were lysed in RIPA buffer (Sigma-Aldrich). Supernatant containing 20 µg of proteins was resolved in SDS-polyacrylamide gel and electroblotted onto PVDF membranes (Millipore, Billerica, MA) using a semidry transfer unit (ATTO, Tokyo, Japan). Immunoblotting was performed with anti-VEGF, MMP-2, Vimentin (Santa Cruz), N-Cadherin, uPA & hnRNP-K (Abcam), phospho-p38MAPK, E-Cadherin (Cell Signaling), HSP27 (StressMarq Biosciences Inc.) and β-actin (Abcam, Cambridge) antibodies. The membranes probed with the first antibodies were excessively washed with TBS-T (Tris-buffered saline-Tween 20) and incubated with secondary horseradish peroxidase (HRP)-conjugated goat anti-mouse or anti-rabbit (Santa Cruz) antibodies. Protein bands were detected using ECL prime substrate (GE Healthcare, CA). Densitometric quantitation of three independent immunoblotting experiments was performed with the Image J software (NIH, Bethesda, MD). Expression level of each of the proteins in control and treated cells was calculated with respect to the β-actin (loading control). All experiments were performed in triplicate.

### Immunofluorescence staining

Cells were cultured as described above. 4 × 10^4^ cells /well were seeded on glass coverslips placed in a 12-well plate for 24 h. Cells were treated with indicated doses of either Wi-A or 3βmWi-A for 24 h and then fixed with pre-chilled methanol on ice for 10 min. PBS-Triton-X-100 (0.2%) for 10 min were used to permeablized the fixed cells followed by blocking with 2% BSA (10 min). The coverslips were probed with antibodies against VEGF, hnRNP-K, Vimentin, N-Cadherin and E-Cadherin at room temperature (1 h) or 4 °C (overnight). Cells were then probed with Alexa Fluor-conjugated secondary antibodies (Molecular Probes, Invitrogen), and counterstained with Hoechst 33258 (Thermo Fisher Scientific). The stained cells were viewed under Zeiss Axioplan 2 microscope and images were captured using a Zeiss AxioCam HRc camera. Acquired images were quantified using ImageJ software, while fluorescence intensity values were normalized with respective controls and represented in percentage values. To examine the distribution and aggregation of Vimentin in Wi-A or 3βmWi-A treated cells, images were acquired under confocal laser scanning microscope (ZEISS LSM 700) and analyzed by IMARIS® software (Bitplane, Switzerland).

### Wound healing assay

Motility of cells was examined using the Wound-healing assay. 2.5 × 10^5^ cells/well were seeded in 6-well plate and allowed to attach to the substratum for next 24 h. Monolayers of cells were wounded on the following day by uniformly scratching the surface with a scrapper tip (20 gauge), followed by washing with PBS and then fresh medium was added. Cells were allowed to proliferate and migrate into the wound for, at least, 24 or 48 h. Movement of the control and treated cells in the scratched area were serially monitored under a phase contrast microscope with a 10 X phase objective (Nikon). Time-lapse live imaging was performed using a microscope (Carl Zeiss MicroImaging, ApoTome) equipped with a Plan-Apochromat 10x/0.45 DICII objective, motorized scanning table and a stage incubator at 37 °C with CO_2_. Images were captured with an AxioCam MR Rev 3 Monochromatic Digital Camera using the AxioVision rel 4.8 software for microscope control and data acquisition. Time-point captured images were processed with image J software adjusting contrast and color threshold options for representation. Percentage covered area was calculated using % area option and crosschecked with analyze particle options using normalized processed images (6–10 randomly captured). The assay was repeated in triplicate in parallel to the Cell Viability Assay.

### Matrigel cell invasion assay

4 × 10^3^ cells were seeded into the upper chamber, coated on the surface with 1/10 dilution of Matrigel (BD BioSciences, Franklin Lakes, NJ), and allowed to migrate to the lower chamber as described in details earlier^41^. Migrated cells were fixed and stained with crystal violet and counted under phase contrast microscopy.

### Reverse-transcription PCR (RT-PCR)

RNA was extracted from control, Wi-A or 3βmWi-A treated cells with Qiagen RNeasy kit. cDNA was synthesized from 2 μg of RNA using the ThermoScript Reverse Transcriptase (ThermoFisher Scientific) following the manufacturer’s protocol. cDNA was subjected to PCR amplification along with gene specific sense and antisense primer set (as follows) using TaKaRa Ex Taq® DNA polymerase, steps consisting of an initial 10 min denaturation step at 95 °C followed by 34 cycles at 95 °C for 45 s, 56 °C for 1 min and 72 °C for 45 s, with final annealing step at 72 °C for 10 min. PCR amplifications were performed using specific primers for (i) hnRNP-K: *Sense*: 5′-ATGAAATTCACCCCCTTTCC-3′ & *Antisense*: 5′-CCCTAGGCTGTGCTCACTTC-3′, (ii) VEGF- *Sense*: 5′-CTGCCCTCAACAAGATGTTTTG-3′ & *Antisense*: 5′-CTATCTGAGCAGCGCTCATGG-3′, (iii) GAPDH: *Sense*: 55-ACCTGACCTGCCGTCTAGAA-3′ & *Antisense*: 5′-TCCACCACCCTGTTGCTGTA-3′. The PCR products were then run on a 1% agarose gel and stained with ethidium bromide for visualization.

### Molecular interaction and docking analysis

Molecular docking and simulations were performed using modules of Schrodinger 2018-2 suite. The structure of proteins and ligands (Wi-A and 3βmWi-A) were prepared using *PrepWizard* and *LigPrep* module respectively^42^. Prepared structures were docked with ligands using Glide extra precision (XP) algorithm^42,43^. The molecular dynamic (MD) simulation of docked complexes was performed to check the stability of ligand interactions at the binding site using Desmond module^42^. All complexes were simulated in a SPC solvated periodic box with 10 Å spacing in Optimized Potential for Liquid Simulations 3 (OPLS3) force field. The solvated system was neutralized, minimized for up to 2000 iterations, heated to 300 K, equilibrated and simulated for a time period ranging between 40 to 50 ns.

i. **Molecular interaction study of Wi-A and 3βmWi-A with hnRNP-K KH3 domain**. Tertiary structure of hnRNP-K KH3 domain with complex to ssDNA was obtained from Protein Data Bank (PDBID: 1ZZI). DNA binding interface of KH3 domain was used to generate grid, so that Wi-A and 3βmWi-A could be screened for their docking potentials at this site^43^. Prepared protein and ligands were then docked using XP docking protocol of Glide module. During docking, hydrogens of aromatic rings were also allowed to form hydrogen bonds.
ii. **Molecular interaction study of Wi-A and 3βmWi-A with monomer, dimer and tetrameric forms of Vimentin**. Since X-ray crystallography structure of full Vimentin is not available so far, we used computational model of Vimentin dimer and tetramer structure generated by Qin *et al*., 2009^44^. In order to investigate the effect of Wi-A and 3βmWi-A, first MD simulation of structure of Vimentin monomer, dimer and tetramer was carried out for 50 ns each. Simulated and stabilized Vimentin monomer, dimer and tetramer molecules were docked with Wi-A and 3βmWi-A around Cys328 residue using Glide extra precision (XP) algorithm.

The stability of inhibitors at the binding site was examined by performing molecular dynamic (MD) simulations of docked complexes. Vimentin monomer-ligand complexes and Vimentin dimer-ligand complexes were simulated for 50 ns each, while Vimentin tetramer-ligand complexes were simulated for 40 ns each. Prime MM-GBSA module of Schrodinger was used to calculate binding energy of protein-ligand complex^42^. Ligplus program was used to study protein-ligand interactions^45^. Root mean square deviation (RMSD), hydrogen bonds analysis and conformational changes over the simulation trajectories of protein-ligand complexes were monitored using VMD version 1.9.4^46^. Images for publication were generated using Pymol molecular graphics system^47^.

**Human MMP Antibody Array.** To examine expression levels of Matrix metalloproteinase family proteins, Human MMP Antibody Array - membrane (#ab134004) were used to determine the total secreted MMP protein quantity in condition media of U2OS cells treated with Wi-A and 3βmWi-A (0.6 μM) following the manufacturer’s protocol.

### Multiplex immunoassay

Bio-Plex Suspension Array System (Bio-Rad, Hercules, CA) was used for multiplex immunoassay profiling. Conditioned medium was collected from control and Wi-A or its methoxy analogue i.e. 3βmWi-A (0.6 μM) treated cells. Concentrations of the cytokines and growth factors in the medium were measured with respect to the standards prepared for each of the factors. Analysis of data was performed with the Bio-Plex Manager software (Bio-Rad), normalized to the control and represented as fold change over control.

### Statistical analysis

All the experiments were carried out in triplicate. Data values were expressed as mean ± SEM of three individual experiment sets. Statistical analyses were carried out using Student’s t-test or nonparametric Mann-Whitney U-test; whichever was applicable. Statistical significance was defined as p-value ≤ 0.05. The *p* value represents * < 0.05, **< 0.01, ***< 0.001.

## Supplementary information

Suppl Information
