# Supplementary material for: 2, 3-Dihydro-3β-methoxy Withaferin-A Lacks Anti-Metastasis Potency: Bioinformatics and Experimental Evidences
Source: Sci Rep. 2019 Nov 22;9:17344. doi: 10.1038/s41598-019-53568-6 (PMC6874665; doi:10.1038/s41598-019-53568-6)
Supplement: Supplementary file 1 — Suppl Information [file 41598_2019_53568_MOESM1_ESM.pdf]

2, 3-Dihydro-3β-methoxy Withaferin-A Lacks Anti-Metastasis Potency: Bioinformatics and Experimental Evidences

Anupama Chaudhary[1], Rajkumar S. Kalra[1], Vidhi Malik[2], Shashank P. Katiyar[2], Durai Sundar[2*], Sunil C. Kaul[1*], and Renu Wadhwa[1*]

Supporting Information:

Legends to Supplementary figures-

Figure S1. RMSD Fluctuations of vimentin protein with time. (**a**) RMSD fluctuations of vimentin monomer (red), dimer (blue) and tetramer (purple) structure taken as control. (**b**) RMSD fluctuations of vimentin monomer protein only for (**i**) control structure (purple), (**ii**) in complex with Wi-A (blue) and (**iii**) in complex with 3βmWi-A (red). (**iv**) RMSD of vimentin monomer-3βmWi-A complex is plotted in green color, which is overlapping with that of protein RMSD. (**c**) RMSD fluctuations of vimentin dimer protein only for (**i**) control structure (purple), (**ii**) in complex with Wi-A (blue) and (**iii**) in complex with 3βmWi-A (red). (**iv**) RMSD of vimentin dimer-3βmWi-A complex is plotted in green color. (**d**) RMSD fluctuations of vimentin tetramer protein only for (**i**) control structure (purple), (**ii**) in complex with Wi-A (blue) and (**iii**) in complex with 3βmWi-A (red). (**iv**) RMSD of vimentin tetramer-3βmWi-A complex is plotted in green color.

Figure S2. Molecular docking of Wi-A and 3βmWi-A with Vimentin. Molecular interactions at vimentin monomer binding site with Wi-A (**a**) and 3βmWi-A (**b**); and molecular binding of Wi-A (**c**) and 3βmWi-A (**d**) with vimentin dimer binding site.

Figure S3. Alignment of vimentin protein sequence with conserved domain sequence of intermediate filament protein family (pfam00038).

Figure S1

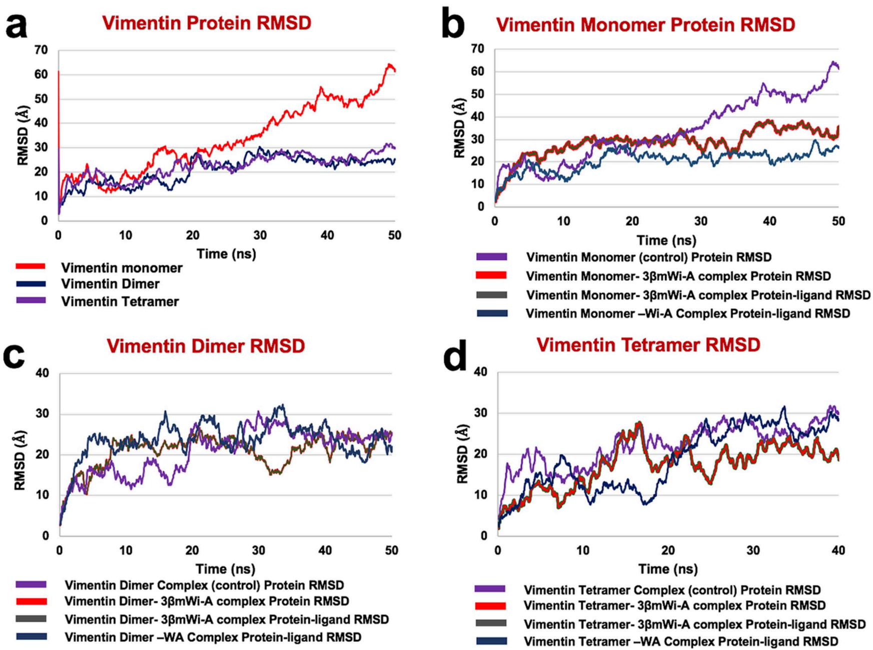
a
Vimentin Protein RMSD
RMSD (Å)
Time (ns)
Vimentin monomer
Vimentin Dimer
Vimentin Tetramer
b
Vimentin Monomer Protein RMSD
RMSD (Å)
Time (ns)
Vimentin Monomer (control) Protein RMSD
Vimentin Monomer- 3βmWi-A complex Protein RMSD
Vimentin Monomer- 3βmWi-A complex Protein-ligand RMSD
Vimentin Monomer –Wi-A Complex Protein-ligand RMSD
c
Vimentin Dimer RMSD
RMSD (Å)
Time (ns)
Vimentin Dimer Complex (control) Protein RMSD
Vimentin Dimer- 3βmWi-A complex Protein RMSD
Vimentin Dimer- 3βmWi-A complex Protein-ligand RMSD
Vimentin Dimer –WA Complex Protein-ligand RMSD
d
Vimentin Tetramer RMSD
RMSD (Å)
Time (ns)
Vimentin Tetramer Complex (control) Protein RMSD
Vimentin Tetramer- 3βmWi-A complex Protein RMSD
Vimentin Tetramer- 3βmWi-A complex Protein-ligand RMSD
Vimentin Tetramer –WA Complex Protein-ligand RMSD

Figure S2

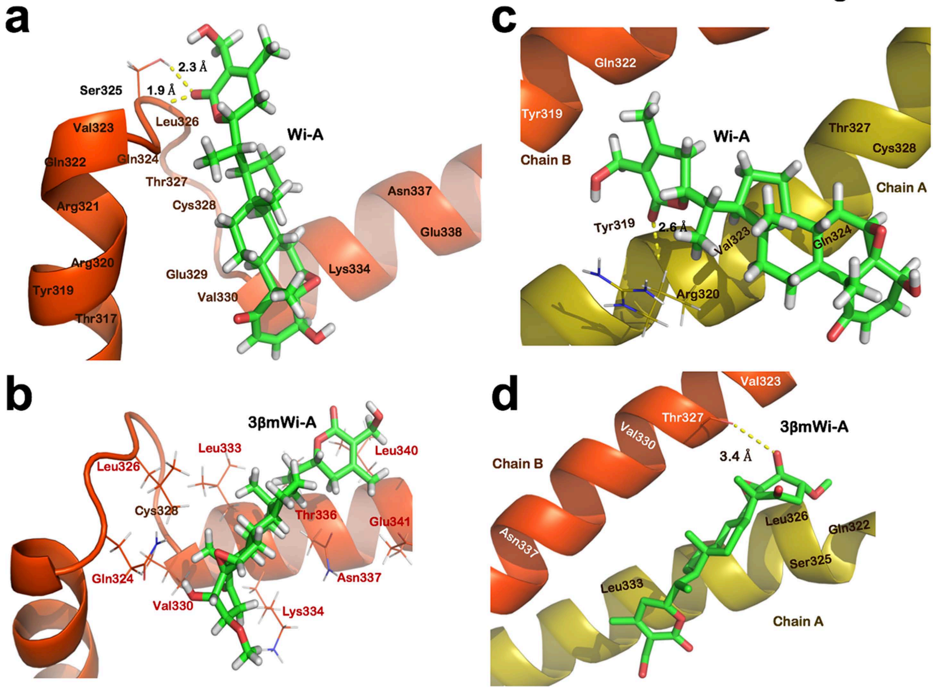
a
2.3 Å
1.9 Å
Ser325
Val323
Leu326
Wi-A
Gln322
Gln324
Thr327
Arg321
Cys328
Asn337
Glu338
Arg320
Glu329
Lys334
Tyr319
Val330
Thr317
b
3βmWi-A
Leu333
Leu340
Leu326
Cys328
Thr336
Glu341
Gln324
Asn337
Val330
Lys334
c
Gln322
Tyr319
Wi-A
Thr327
Cys328
Chain B
Chain A
Tyr319
2.6 Å
Val323
Gln324
Arg320
d
Val323
Thr327
3βmWi-A
Val330
Chain B
3.4 Å
Leu326
Gln322
Asn337
Ser325
Leu333
Chain A

Figure S3

[Superfamily] cl25641 (PSSMID 330462) Intermediate filament protein ;Intermediate filament protein.
CD-length: 313 E-value: 7.31e-139 Bitscore: 405

```
                           10        20        30        40        50
                   ....*....|....*....|....*....|....*....|....*....|
Vimentin       102 NEKVELQELNDRFANYIDKVRFLEQQNKILLAELEQLKGQ---GKSRLGD 148
Cdd:pfam00038    1 NEKEQLQELNDRLASYIDKVRFLEQQNKDLETKISELRQKkgaEPSRLYS 50
                           60        70        80        90       100
                   ....*....|....*....|....*....|....*....|....*....|
Vimentin       149 LYEEEMRELRRQVDQLTNDKARVEVERDNLAEDIMRLREKLQEEMLQREE 198
Cdd:pfam00038   51 LYEREIRDLRRQLDQLTVERARLQLEIDNLRLAAEDFRQKYEDELNLRQS 100
                          110       120       130       140       150
                   ....*....|....*....|....*....|....*....|....*....|
Vimentin       199 AENTLQSFRQDVDNASLARLDLERKVESLQEEIAFLKKLHEEEIQELQAQ 248
Cdd:pfam00038  101 AEADLVGLRKDLDEATLARVDLEMKVESLQEELAFLKKNHEEEVRELQSQ 150
                          160       170       180       190       200
                   ....*....|....*....|....*....|....*....|....*....|
Vimentin       249 IQEQHVQIDVDVS-KPDLTAALRDVRQQYESVAAKNLQEAEEWYKSKFAD 297
Cdd:pfam00038  151 VQDTQVNVEMDAArKLDLTSALAEIRAQYEEIAAKNREEAEEWYQSKLEE 200
                          210       220       230       240       250
                   ....*....|....*....|....*....|....*....|....*....|
Vimentin       298 LSEAANRNNDALRQAKQESTEYRRQVQSLTCEVDALKGTNESLERQMREM 347
Cdd:pfam00038  201 LQQAAARNGDALRSAKEEITELRRQIQSLEIELQSLKKQKASLERQLAET 250
                          260       270       280       290       300
                   ....*....|....*....|....*....|....*....|....*....|
Vimentin       348 EENFAVEAANYQDTIGRLQDEIQNMKEEMARHLREYQDLLNVKMALDIEI 397
Cdd:pfam00038  251 EERYELQLADYQDLISELEAELQQIRQEMARQLREYQELLNVKLALDIEI 300
                          310
                   ....*....|...
Vimentin       398 ATYRKLLEGEESR 410
Cdd:pfam00038  301 ATYRKLLEGEECR 313
```
